# Pan-Cancer Analysis Reveals SH3TC2 as an Oncogene for Colorectal Cancer and Promotes Tumorigenesis via the MAPK Pathway

**DOI:** 10.3390/cancers14153735

**Published:** 2022-07-31

**Authors:** Chengzhi Huang, Hui Yi, Yue Zhou, Qing Zhang, Xueqing Yao

**Affiliations:** 1 School of Medicine, South China University of Technology, Guangzhou 510006, China; huangchengzhi93@hotmail.com; 2 Department of Gastrointestinal Surgery, Department of General Surgery, Guangdong Provincial People’s Hospital, Guangdong Academy of Medical Sciences, Guangzhou 510080, China; 201921047477@mail.scut.edu.cn (H.Y.); zhouyuesjd@163.com (Y.Z.); 3 Department of General Surgery, Guangdong Provincial People’s Hospital Ganzhou Hospital (Ganzhou Municipal Hospital), Ganzhou 341000, China; 4 School of Biology and Biological Engineering, South China University of Technology, Guangzhou 510006, China; 5 Department of Pharmacology, The First People’s Hospital of Zhaoqing, Zhaoqing 526000, China; 6 The Second School of Clinical Medicine, Southern Medical University, Guangzhou 510515, China; 7 Department of Gastrointestinal and Anorectal Surgery, The First People’s Hospital of Zhaoqing, Zhaoqing 526000, China

**Keywords:** pan-cancer analysis, SH3TC2, tumorigenesis, colorectal cancer, MAPK pathway

## Abstract

**Simple Summary:**

SH3 domain and tetrapeptide repeat 2 (SH3TC2) is a protein-encoding gene and has previously been described as a critical signaling hub for neurological disorders, but no systematic analysis of SH3TC2 is available in cancer research. We analyzed SH3TC2 in various kinds of cancer to find its tumorigenic role in one or more specific cancers and further explored the mechanism of *SH3TC2* in colorectal cancer (CRC). Our research revealed that higher expression of *SH3TC2* indicated poor disease-free survival and promoted CRC progression and invasion via the MAPK signaling pathway.

**Abstract:**

SH3 domain and tetrapeptide repeat 2 (SH3TC2) is a protein-encoding gene and has previously been described as a critical signaling hub for neurological disorders. Although increasing evidence supports a vital role of SH3TC2 in the tumorigenesis of various kinds of cancer, no systematic analysis of SH3TC2 is available. The function and mechanism of *SH3TC2* in other cancers remain unknown. Thus, this study aimed to analyze SH3TC2 in various kinds of cancer to find its tumorigenic role in one or more specific cancers. In the current study, we analyzed the expression level and prognostic value of *SH3TC2* in different tumors in the TCGA-GTEx pan-cancer dataset. Subsequently, the prognostic role and mechanism of *SH3TC2* in colorectal cancer (CRC) were further explored via clinical samples and in vitro and in vivo experiments. We observed differential expression of *SH3TC2* in colon adenocarcinoma (COAD), acute myeloid leukemia (LAML), READ (rectum adenocarcinoma), SKCM (skin cutaneous melanoma), and TGCT (testicular germ cell tumors). Subsequently, *SH3TC2* showed a significant effect on the clinical stage and prognostic value in CRC, LAML, and SKCM. Moreover, we found in the TCGA database and seven GEO datasets that *SH3TC2* was significantly highly expressed in tumor tissue. Through enrichment analysis of *SH3TC2* and its co-expressed genes, we found that *SH3TC2* may play a role in the MAPK signaling pathway. Correlation analysis indicated that *SH3TC2* was significantly associated with multiple key factors in the MAPK signaling pathway. Additionally, higher expression of SH3TC2 was found in tumor tissue in our cohort including 40 CRC patients. Overexpression of SH3TC2 may imply poor prognosis. Knockdown of *SH3TC2* significantly inhibited tumor invasion, migration, and proliferation. More importantly, knockdown of *SH3TC2* inhibited tumor growth in a CRC mouse model. The study preliminarily conducted a pan-cancer study of *SH3TC2* and further explored the mechanism of *SH3TC2* in CRC. Our research revealed that higher expression of *SH3TC2* may promote CRC progression and invasion via the MAPK signaling pathway.

## 1. Introduction

Cancers bring heavy burdens to global health. According to global cancer burden data, the number of new cancer patients worldwide in 2020 was 19.3 million, and the number of deaths due to cancer approached 10 million [1]. Colorectal cancer (CRC) is the third most common cancer and has high recurrence and mortality [1,2,3,4]. Tumorigenesis, recurrence, and tumor metastasis are closely related to epigenetic dysregulation [5,6,7,8]. Therefore, there is an unmet clinical and basic medicine demand to determine the underlying molecular oncogenic alterations leading to cancer recurrence and mortality, and to explore novel therapeutic strategies to improve CRC patient survival.

SH3 domain and tetratricopeptide repeats 2 (*SH3TC2*) is a protein-coding gene that encodes a protein with two N-terminal Src homology 3 domains and 10 tetratricopeptide repeat motifs [9,10]. SH3TC2 plays a key role in neurological diseases such as Charcot-Marie-Tooth disease type 4C [11,12]. Previous research reported that *SH3TC2* gene mutations cause autosomal recessive type 4C peroneal muscular dystrophy [13]. In addition, researchers found that expression of *SH3TC2* in the cerebellum was regulated by single nucleotide polymorphisms, which may be involved in the pathogenesis of schizophrenia [14].

Furthermore, *SH3TC2* has been reported to play an important role in tumorigenesis and tumor progression. Yu et al. found that *SH3TC2* was highly expressed in LAML and was associated with the most common tyrosine kinase 3 mutations in LAML, leading to speculation that *SH3TC2* may be a prognostic marker for LAML [15]. Doecke et al. identified *SH3TC2* as a DNA methylation-localized gene associated with a high prevalence of cancer by analyzing the methylation data of 15 cancer types in The Cancer Genome Atlas (TCGA) database [16].

The mitogen-activated protein kinase (MAPK) signaling pathway is one of the important pathways in the eukaryotic signaling network and is involved in various biological processes, such as cell proliferation, differentiation, and apoptosis [17,18,19,20,21]. MAPK is a group of evolutionarily conserved serine–threonine kinases, including four subfamilies, namely, extracellular signal-regulated protein kinase (ERK), p38 mitogen-activated protein kinase (p38 MAPK), c-Jun N-terminal kinase (JNK), and extracellular signal-regulated kinase 5 (ERK5) [22,23,24,25,26,27]. These kinases are involved in multiple signal transduction pathways and perform a wide variety of functions. Among them, the most widely studied is ERK1/ERK2 kinase, which is involved in the RAS-Raf-MEK 1/2-ERK 1/2 pathway, which is closely related to the occurrence of multiple cancers [28,29,30,31]. The MAPK signaling pathway has been implicated in the development of many human diseases, including Alzheimer’s disease [32,33,34], Parkinson’s disease, and various kinds of cancers [35,36]. The MAPK signaling pathway is related to the occurrence, progression, and metastasis of colorectal cancer [37,38]. Li et al. found that Mex3a promotes oncogenesis through the RAP1/MAPK signaling pathway in colorectal cancer [17]. BRAF gene mutation is a common mutation type in CRC, and a combination therapy strategy for MAPK pathway blockade brings hope for the treatment of metastatic CRC patients with BRAF v600e mutation [39,40,41,42].

In the present study, we comprehensively explored the expression profile and prognostic value of SH3TC2 in various kinds of solid tumors via the TCGA database and found that SH3TC2 was more highly expressed in multiple cancer types and was closely related to cancer patient survival. After integrating the analysis from different databases, we demonstrated that SH3TC2 was more highly expressed in CRC and positively related to poor disease-free survival (DFS). In vitro and in vivo experiments revealed that knockdown of *SH3TC2* significantly inhibited tumor proliferation and tumorigenesis. Knockdown of *SH3TC2* in cell lines significantly inhibited MAPK pathways via MAPK8, 9, 13, and 14. Combining comprehensive bioinformatics analysis and in vitro and in vivo experiments, we are the first to report that SH3TC2 is a novel oncogene and may act as a potential prognostic biomarker and therapeutic target for CRC. 

## 2. Materials and Methods

### 2.1. Data Sources

The standardized pan-cancer dataset (TCGA-GTEx) was acquired from the open-access UCSC (https://xenabrowser.net/) database (accessed on 15 March 2022) and consists of screened tumor and normal samples from primary solid tumors. Subsequently, we filtered the samples with an expression level of 0, excluded cancer types with fewer than 3 samples, and finally obtained the expression data of 27 cancer species. The cancer names and their corresponding abbreviations for all cancer types are listed in Table S1.

### 2.2. SH3TC2 Expression Pattern in Pan-Cancer

The expression levels of *SH3TC2* in tumor and normal samples of 27 cancer types were analyzed by the limma package [43] in R software, and the significance of differences was assessed using the unpaired Student’s *t* test. Subsequently, we further verified the expression levels of *SH3TC2* in different tumors through the Gene Expression Profiling Interactive Analysis (GEPIA) database (http://gepia.cancer-pku.cn/) (accessed on 15 March 2022) [44].

### 2.3. Prognostic Analysis of SH3TC2 in Different Tumors

This study analyzed the effect of *SH3TC2* on the disease-free survival (DFS) and overall survival (OS) of patients with different tumors via the GEPIA database. All patients of each cancer type were divided into high-expression groups and low-expression groups according to the median value of the *SH3TC2* expression level.

### 2.4. SH3TC2 Expression Levels in CRC

The expression values of *SH3TC2* in the TCGA database and Gene Expression Omnibus (GEO) database, including the GSE32323, GSE44076, GSE21815, GSE31279, GSE41657, GSE83889, and GSE87211 datasets, were extracted and log_2_ transformed. We analyzed the expression difference of *SH3TC2* between tumor samples and normal samples in the TCGA database and 7 GEO datasets. The TCGA and GEO databases were assessed on 1 March 2022. 

### 2.5. Correlation between SH3TC2 Expression and Clinical Stage in Colorectal Cancer

We analyzed the expression levels of *SH3TC2* in normal tissue samples, tumor tissue samples, and metastatic tissue samples of colon cancer microarray data in the GEO database through the TNM plot database (https://tnmplot.com/) (accessed on 27 May 2022) [45]. Furthermore, we analyzed the relative expression levels of *SH3TC2* in the different clinical stages of CRC samples from the TCGA database.

### 2.6. Correlation between SH3TC2 Expression and Immune Cell Infiltration

In this study, the Cibersoft, Timer, and MCPcounter algorithms were used to calculate the infiltration levels of various immune cells in CRC tumor samples (TCGA-COAD (*n* = 282), TCGA-READ (*n* = 91)) from the TCGA database. The deconvo_Cibersoft algorithm of the language IOBR [46] software package calculates 22 types of immune cell infiltration scores, the Timer algorithm calculates 6 types of immune cell infiltration scores, and the MCPcounter algorithm calculates 10 types of immune cell infiltration scores. Then, we analyzed the Pearson correlation coefficient between SH3TC2 expression and the above immune cell infiltration scores by the Corr.test function of the psych package in R language.

### 2.7. The Co-Expressed Genes of SH3TC2 and Functional Enrichment Analysis

The co-expressed genes of *SH3TC2* in colon cancer (TCGA-COAD) and rectal cancer (TCGA-READ) samples in the TCGA database were obtained through the “Similar Genes”. Then, we screened the top 30 co-expressed genes according to the Pearson correlation coefficient (PCC). Subsequently, we performed Gene Ontology (GO) and Kyoto Encyclopedia of Genes and Genomes (KEGG) enrichment analyses on the co-expressed genes by the clusterProfiler package of R software [47] to explore the potential biological functions of *SH3TC2*.

### 2.8. Clinical Specimens and Immunohistochemistry (IHC) Analysis

Surgical resection samples were obtained from 40 CRC patients who underwent surgical resection at Guangdong Provincial People’s Hospital (Guangdong Academy of Medical Sciences). All of the patients included were undergoing surgical resection and were pathologically diagnosed with adenocarcinoma. All enrolled CRC patients provided written consent, and collection of the clinical sample was approved by the Institutional Review Board of Guangdong Provincial People’s Hospital under approval number GDREC2019504H(R2).

To further analyze the potential association between SH3TC2 and CRC, IHC analysis was performed as previously described [48,49,50]. The list of antibodies and dilution fold can be found in Supplementary Materials Table S2. The procedure for IHC analysis was like our previously reported study [49]. The score of positive cells was defined as 0 (<10%), 1 (10–25%), 2 (25–50%), and 3 (>50%), and the score of staining intensity was defined as 0 (negative staining), 1 (weak staining), 2 (moderate staining), and 3 (strong staining). The final score was calculated as the sum of the score of positive cells and staining intensity. High expression of SH3TC2 was identified as 4–6 on IHC score, and low expression was identified as 0–3.

### 2.9. Cell Lines and Transfection

*SH3TC2* gene expression was investigated via the Cancer Cell Line Encyclopedia (CCLE) database (https://sites.broadinstitute.org/ccle) (accessed on 3 March 2022). Then, the human CRC cell lines HCT116, SW480, and LOVO were purchased from iCell Bioscience (Shanghai, China). The included cell lines were incubated with RPMI-1640 medium (G4530, Servicebio, Wuhan, China) containing 10% fetal bovine serum (900–108, GeminiBio, West Sacramento, CA, USA) at 37 °C in a humidified incubator containing 5% CO_2_. SH3TC2 small interfering RNA (siRNA) was synthesized by Sangon Biotech (Shanghai, China). Three SH3TC2 siRNAs were designed in siDirect version 2.0 (http://sidirect2.rnai.jp/) (accessed on 4 March 2022) [51], and the sequence can be found in Table S3. The siRNAs were transfected into the cells by Lipofectamine 3000 (Invitrogen, Waltham, MA, USA) following the manufacturer’s instructions. For the stable slicing of SH3TC2 in cell lines, the slicing sequence was packed into LV3 lentivirus with the resistance gene purine. Stable transfection was selected by puromycin (BL528A, Biosharp, Shanghai, China) for at least 7 days.

### 2.10. Quantitative Real-Time Polymerase Chain Reaction (qRT-PCR)

We performed qRT-PCR analysis as previously described [48,49,50]. Briefly, total RNA was extracted by Total RNA Isolation Reagent (BS258A, Biosharp, Shanghai, China) and reverse transcribed into complementary DNA (cDNA) by the First-strand cDNA Synthesis Kit (K1072, Apexbio, Huston, TX, USA). Expression of genes was quantified by the SYBR Green PCR Mix (BL697A, Biosharp, Shanghai, China) on the LightCycler 480 (Roche, Basel, Switzerland) instrument. The primer sequences can be found in Table S4.

### 2.11. Western Blot

Western blot analysis was utilized to examine the protein expression level of SH3TC2 and its related proteins in CRC cell lines. The experiments were performed as previously described [49,50]. The list of antibodies and dilution fold can be found in Supplementary Materials Table S2. The original Western blot and statistical analysis can be found in Figures S5–S8 and Tables S6–S9. Relative gray intensity analysis was performed by ImageJ software.

### 2.12. Transwell Analysis

Cell migration was analyzed by Transwell analysis. Briefly, a total of 500 μL of RPMI-1640 medium containing 10% fetal bovine serum was added to the lower chamber of the Transwell plate. The cells were seeded into the upper chamber of the plate with serum-free medium. After incubation for 72 h, the migratory cells were fixed and stained with 0.1% crystal violet solution (PH1322, Phygene, Fuzhou, China). The migrated cells were observed under a light microscope (Eclipse Ni-U, Nikon, Tokyo, Japan) and were analyzed by ImageJ software.

### 2.13. Cell Viability Analysis

To investigate the cell viability of the cell lines, the cell counting kit 8 (CCK-8) detection kit (K1018, Apexbio, Huston, TX, USA) was utilized in our study. Briefly, a total of 1500 cells/well in 100 μL complete cell culture medium was seeded in a 96-well plate. After incubation of 0, 24, 48, or 72 h, 10 μL of CCK-8 solution was added to the wells. Absorbance in 450 nm light was assessed on a microplate reader (Synergy H1, Bio-Tek, Winooski, VT, USA). 

### 2.14. Cell Apoptosis Assays

To detect cell apoptosis after different treatments, a flow cytometer assay was performed with the cell apoptosis kit (E-CK-A211, Elabscience, Wuhan, China) following the manufacturer’s instruction. After staining with Annexin-FITC and PI, flow cytometer (BD FACSVerse, BD Bioscience, Franklin Lakes, NJ, USA) was utilized to collect the cell samples. The raw data were analyzed by FlowJo software version 10.6 (BD Bioscience, Franklin Lakes, NJ, USA). Anisomycin was purchased from Beyotime Biotechnology, Shanghai, China (SC0132). 

### 2.15. In Vivo Analysis

Nude mice (6–8 weeks old) were randomly allocated to two groups. All mice were purchased from Guangdong GemPharmatech (Foshan, China) and bred in specific-pathogen-free (SPF) conditions. To establish the CRC model in nude mice, 5 × 10^6^ HCT116 negative control (HCT116 shNC) cells or HCT116 stable slicing SH3TC2 (HCT116 shSH3TC2) cells were subcutaneously injected into the right flank. Tumor volume was measured every two days and defined as follows: volume = length × width^2^ × 0.5 mm^3^. The study was approved by the Ethics Committee of Guangdong Provincial People’s Hospital (Guangdong Academy of Medical Sciences) under approval number GDREC2019506A. 

### 2.16. Statistical Analysis

The included bioinformatics and statistical analyses were performed using R software version 4.1.3 (http://www.r-project.org/, accessed on 15 March 2022), GraphPad Prism 9.3 (Dotmatics, San Diego, CA, USA), and Stata 15.0 (Stata Corp, College Station, TX, USA). Data were presented as mean ± standard deviation (SD). The difference between groups was analyzed by Student’s *t*-test, unpaired *t*-test, or one-way ANOVA. Prognostic analysis was performed by log-rank test. Experiments were performed in triplicate, and *p* < 0.05 was considered statistically significant.

## 3. Results

### 3.1. Expression Levels and Prognostic Value of SH3TC2 in Human Cancers

**Figure 1:**
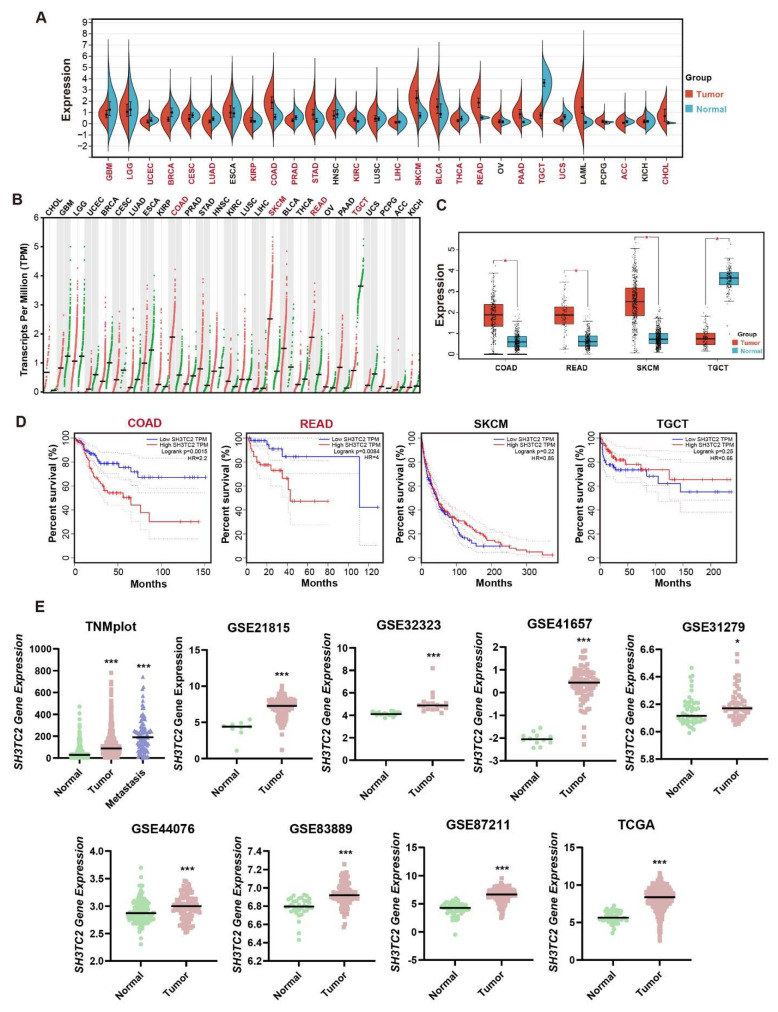
Pan-cancer analysis revealed the expression levels and prognostic value of SH3TC2 in human cancers. (**A**) Comparison of SH3TC2 expression in tumor tissue and normal tissue in the TCGA database. The red-highlighted cancer names indicate significance (*p* < 0.05). The abbreviations can be found in Table S2. (**B**) Comparison of SH3TC2 expression in tumor tissue and normal tissue in the GEPIA database. The red-highlighted cancer names indicate that SH3TC2 is differentially expressed in tumor tissue and normal tissue based on screening of the GEPIA and TCGA databases. (**C**) Comparison of SH3TC2 expression in tumor tissue and normal tissue in specific cancers, including COAD, READ, SKCM, and TGCT. Analysis revealed that SH3TC2 is upregulated in CRC (COAD and READ) and SKCM but downregulated in TGCT. (**D**) Prognostic analysis of the GEPIA database revealed that high SH3TC2 expression is correlated with poor DFS in CRC patients. No statistical significance was found in SKCM and TGCT. (**E**) Analysis of the TNMplot database, TCGA database, and 7 GEO datasets indicated that SH3TC2 is upregulated in tumor tissue in CRC. Statistical analysis was compared to the control or normal groups: * *p* < 0.05; *** *p* < 0.001.

The expression levels of *SH3TC2* in the 27 types of cancers are shown in Figure 1A. The results showed that the expression of SH3TC2 was significantly higher in several cancers compared to normal tissues, including kidney renal papillary cell carcinoma (KIRP) (*p* < 0.01), COAD (*p* < 0.0001), stomach adenocarcinoma (STAD) (*p* < 0.0001), kidney renal clear cell carcinoma (KIRC) (*p* < 0.0001), skin cutaneous melanoma (SKCM) (*p* < 0.0001), bladder urothelial carcinoma (BLCA) (*p* < 0.0001), READ (*p* < 0.0001), pancreatic adenocarcinoma (PAAD) (*p* < 0.0001), and cholangiocarcinoma (CHOL) (*p* < 0.001). In addition, *SH3TC2* expression was significantly downregulated in 12 cancers, including glioblastoma multiforme (GBM) (*p* < 0.0001), brain lower grade glioma (LGG) (*p* < 0.001), uterine corpus endometrial carcinoma (UCEC) (*p* < 0.001), breast invasive carcinoma (BRCA) (*p* < 0.0001), cervical squamous cell carcinoma and endocervical adenocarcinoma (CESC) (*p* < 0.05), lung adenocarcinoma (LUAD) (*p* < 0.0001), prostate adenocarcinoma (PRAD) (*p* < 0.0001), liver hepatocellular carcinoma (LIHC) (*p* < 0.05), thyroid carcinoma (THCA) (*p* < 0.0001), testicular germ cell tumors (TGCT) (*p* < 0.0001), uterine carcinosarcoma (UCS) (*p* < 0.0001), and adrenocortical carcinoma (ACC) (*p* < 0.0001). Subsequently, expression of SH3TC2 in the TCGA-GTEx database was verified by the GEPIA database (Figure 1B). Based on our analysis results and the results from the GEPIA database, SH3TC2 was differentially expressed in COAD, READ, SKCM, and TGCT (Figure 1C).

Then, we further focused on COAD, READ, SKCM, and TGCT. We analyzed the effect of *SH3TC2* on the disease-free survival (DFS) of COAD, READ, SKCM, and TGCT patients in the GEPIA database. The results showed that high *SH3TC2* expression predicted worse DFS for colon cancer patients (hazard ratio, HR = 2.20, *p* = 0.0015) and rectal cancer patients (HR = 4.00, *p* = 0.0084), while the level of SH3TC2 expression in SKCM (HR = 0.86, *p* = 0.22) and TGCT (HR = 0.66, *p* = 0.25) had no significant effect on DFS (Figure 1D).

However, higher expression of *SH3TC2* in colon cancer, rectal cancer, or CRC did not imply poor prognosis in the overall survival (OS) of cancer patients (Figure S1). The results of bioinformatics analysis of the GEPIA database implied that higher expression of SH3TC2 in tumor tissue indicates a poor prognosis in CRC patients. However, due to the conflicting results in OS analysis, the prognostic value of *SH3TC2* still needs to be further validated.

Additionally, we found that the relative expression level of *SH3TC2* in CRC samples in the TCGA database was significantly higher than that in normal samples (*p* < 0.0001). Furthermore, we observed high expression levels of *SH3TC2* in CRC samples in the GSE32323 (*p* < 0.001), GSE44076 (*p* < 0.001), GSE21815 (*p* < 0.001), GSE31279 (*p* < 0.05), GSE41657 (*p* < 0.001), GSE83889 (*p* < 0.001), and GSE87211 (*p* < 0.001) datasets (Figure 1E). This result indicates that SH3TC2 may act as a novel oncogene for CRC.

**Figure 2:**
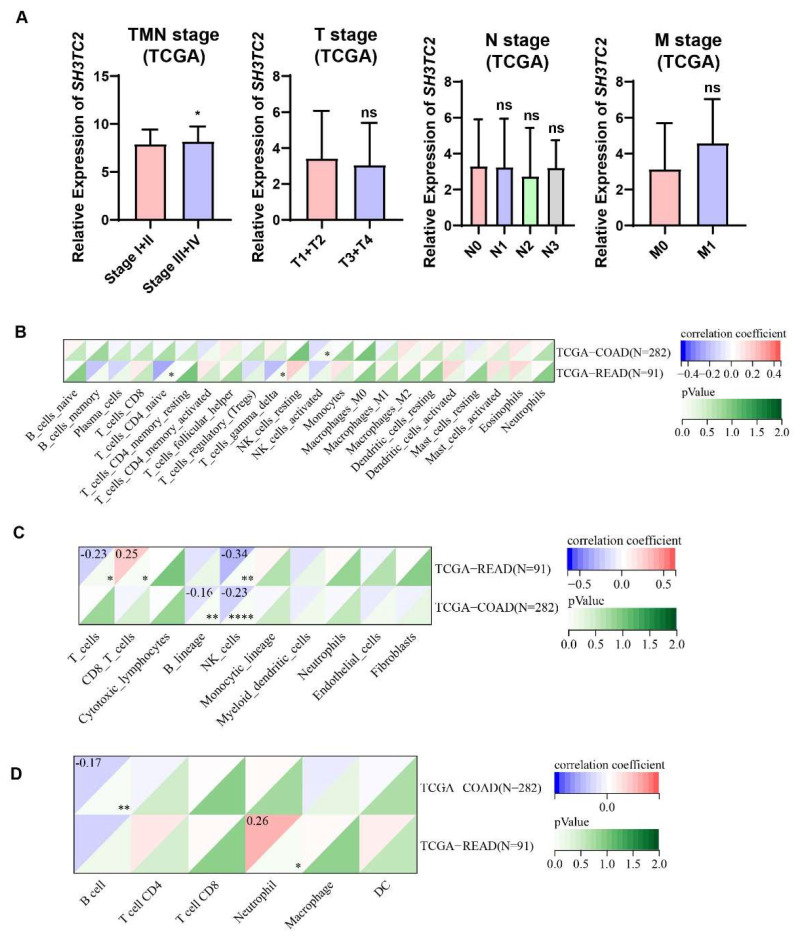
Analysis of TCGA database revealed the expression of SH3TC2 and clinical characteristics in CRC. (**A**) Advanced CRC patients had higher expression of SH3TC2. However, expression of SH3TC2 did not correlate with the T, N, or M stages. Cibersoft (**B**), MCPCounter (**C**), and TIMER (**D**) analyses revealed that the expression of SH3TC2 may correlate with T-cell infiltration. Statistical analysis was compared to the control or normal groups: ns, not significant; * *p* < 0.05; ** *p* < 0.01; **** *p* < 0.001.

We evaluated the expression of *SH3TC2* in the clinical stage of colon cancer in gene chip data through the TMNplot database. The results showed that SH3TC2 was differentially expressed between normal and tumor tissues, between tumor and metastatic tissues, and between normal and metastatic tissues (Figure 1E). Furthermore, we found that the relative expression of SH3TC2 in CRC samples in the TCGA database was significantly correlated with the TNM stage (Figure 2A). However, expression of SH3TC2 was not independently related to the T stage, N stage, or M stage (Figure 2A).

### 3.2. Correlation between SH3TC2 Expression and Immune Cell Infiltration Levels

Through the Cibersoft algorithm, we observed that *SH3TC2* correlated with NK_cells_activated immune infiltration in COAD (R = −0.12, *p* = 0.042) and correlated with the immune infiltration level of T_cells_CD4_naive (R = −0.28, *p* = 0.007) and T_cells_gamma_delta (R = −0.22, *p* = 0.033) in READ (Figure 2B). Using the MCPcounter algorithm, we found that SH3TC2 was associated with B_lineage (R = −0.16, *p* = 0.008) and NK_cells (R = −0.23, *p* < 0.001) infiltration levels in COAD and with the infiltration levels of T_cells (R = −0.23, *p* = 0.030), CD8_T_cells (R = 0.25, *p* = 0.016), and NK_cells (R = −0.34, *p* = 0.001) in READ (Figure 2C). Using the TIMER algorithm, we observed that SH3TC2 correlated with the infiltration levels of B cells (R = −0.17, *p* = 0.005) in COAD and the infiltration levels of neutrophils (R = 0.26, *p* = 0.012) in READ (Figure 2D).

### 3.3. Clinical Specimen Analysis Revealed That Higher Expression of SH3TC2 Indicated Poor Prognosis

**Figure 3:**
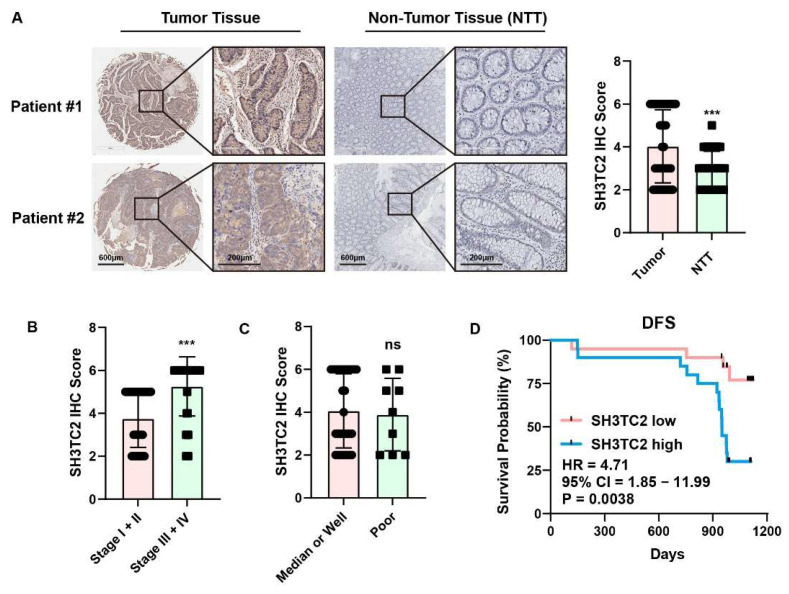
Overexpressed SH3TC2 indicated poor clinical prognosis. (**A**) Representative SH3TC2 IHC staining of clinical specimens. In our cohort, expression of SH3TC2 was significantly upregulated in tumor tissues compared with nontumor tissues (NTTs). (**B**) SH3TC2 is upregulated in advanced CRC patients (TNM III and IV stages). (**C**) Expression of SH3TC2 is not related to tumor differentiation. (**D**) Kaplan-Meier analysis of CRC patients with high or low expression of SH3TC2. Patients with higher SH3TC2 expression had poor DFS. Statistical analysis was compared between two groups: ns, not significant; *** *p* < 0.001.

A total of 40 CRC patients were enrolled in our study. Tumor tissue and non-tumor tissue (NTT) specimens were collected after surgical resection and analyzed after IHC staining. In our cohort, expression of SH3TC2 protein was significantly higher in tumor tissue than in NTT tissue (Figure 3A). Expression of SH3TC2 was also significantly correlated with TNM clinical stage in our cohort (Figure 3B). However, expression of SH3TC2 protein was not related to tumor differentiation (Figure 3C). The correlation between SH3TC2 expression and the clinicopathologic features of CRC patients in our cohort can be found in Table S5. Furthermore, we investigated the prognostic value of SH3TC2 expression in CRC. As shown in Figure 3D, in our cohort, we found that higher expression of SH3TC2 in tumor tissue indicated poor DFS of CRC patients (HR = 4.71, 95% CI = 1.85–11.99, *p* = 0.0038).

### 3.4. Downregulated SH3TC2 Expression Inhibits Tumorigenesis In Vitro

**Figure 4:**
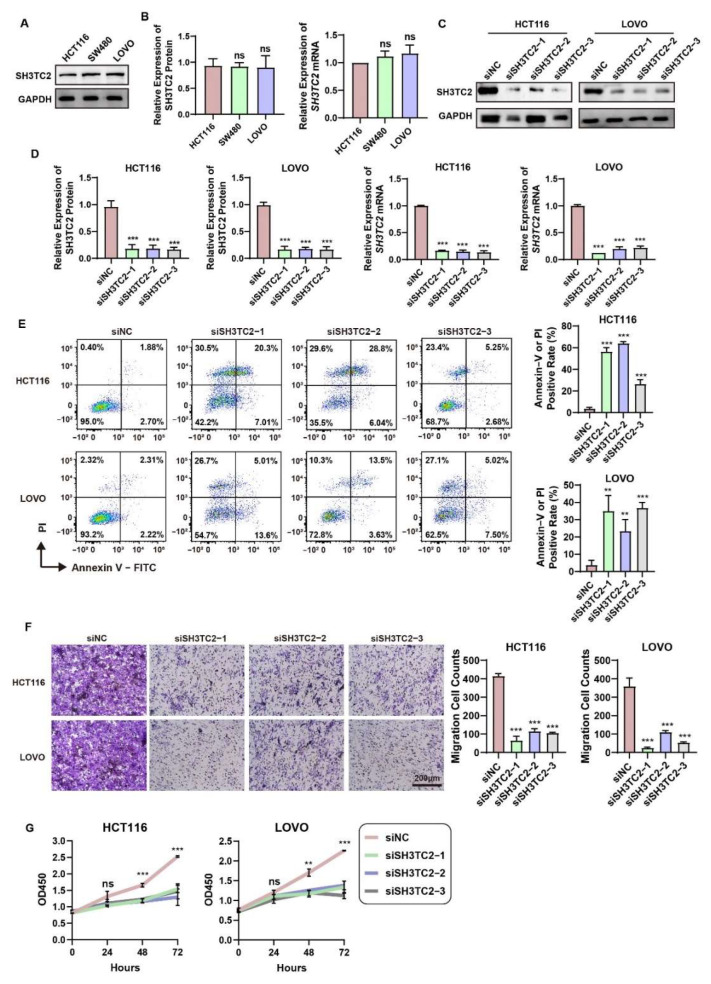
Downregulation of SH3TC2 inhibited tumor progression and promoted cell apoptosis. (**A**) Comparison of SH3TC2 expression in three CRC cell lines via Western blotting. (**B**) The qRT-PCR and quantitative Western blotting analyses revealed no expression difference among the three cell lines. (**C**) Expression of SH3TC2 was significantly downregulated in HCT116 and LOVO cell lines after transfection with three SH3TC2 siRNAs. (**D**) The qRT-PCR and quantitative Western blotting analyses revealed that SH3TC2 was significantly downregulated in HCT116 and LOVO cells after siRNA transfection. (**E**) Downregulation of SH3TC2 significantly improved cell apoptosis. (**F**) Downregulation of SH3TC2 significantly decreased cell invasion ability. (**G**) Downregulation of SH3TC2 significantly decreased cell proliferation. The statistical analysis was compared to the control or normal groups: ns, not significant; ** *p* < 0.01; *** *p* < 0.001.

We explored the expression of *SH3TC2* in CRC cell lines via the CCLE database (Figure S2). Then, we further investigated the expression of SH3TC2 in HCT116, SW480, and LOVO cell lines through Western blotting and qRT-PCR analysis. As shown in Figure 4A,B, expression of SH3TC2 protein and mRNA was similar in the cell lines HCT116, SW480, and LOVO, and no statistical significance was found. The results were consistent with the analysis of the CCLE database. Consequently, HCT116 and LOVO cells were selected for further analysis. After the transfection of three siRNAs, the expression of SH3TC2 protein and mRNA was significantly downregulated (Figure 4C,D), which means the siRNA transfection was effective. As a result, the sequence of siSH3TC2-3 was further utilized to construct the short hairpin RNA (shRNA) and lentivirus for stable transfection.

The results of the flow cytometry assay revealed that downregulation of SH3TC2 might significantly increase cell apoptosis in HCT116 and LOVO cell lines (Figure 4E). The cell migration assay displayed an inhibitory effect on HCT116 and LOVO cell lines due to SH3TC2 downregulation (Figure 4F). The CCK-8 assay revealed that the viability of siSH3TC2-transfected HCT116 and LOVO cell lines was significantly lower than that of the negative control cell lines (Figure 4G).

### 3.5. Coexpressed Genes of SH3TC2 and Functional Enrichment Analysis

**Figure 5:**
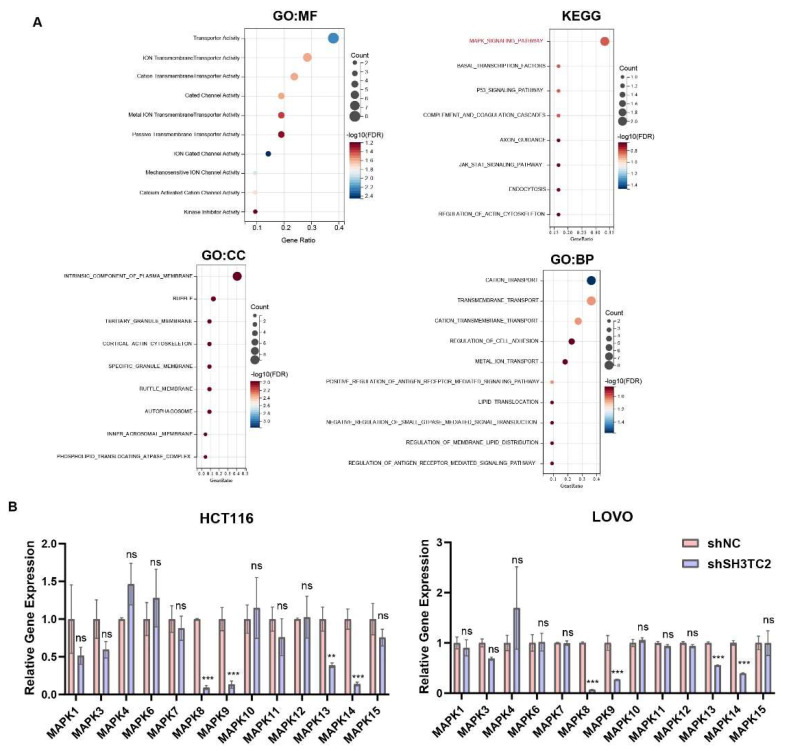
Genes co-expressed with SH3TC2 and functional enrichment analysis. (**A**) GO and KEGG functional enrichment analyses were performed on *SH3TC2* and co-expressed genes in CRC. Bioinformatics analysis implied that the MAPK pathway was most likely correlated with the expression of SH3TC2. (**B**) The qRT-PCR analysis of key points of MAPK pathway regulators after the stable transfection of shSH3TC2 in HCT116 and LOVO cells. The results implied that MAPK8, MAPK9, MAPK13, and MAPK14 were significantly regulated after silencing SH3TC2. Statistical analysis was compared to the control group: ns, not significant; ** *p* < 0.01; *** *p* < 0.001.

GO and KEGG functional enrichment analyses were performed on *SH3TC2* and coexpressed genes (Figure 5A). According to the thresholds of *p* < 0.05 and FDR < 0.25 in KEGG functional enrichment analyses, eight significantly enriched pathways were obtained, including the MAPK signaling pathway, basal transcription factors, the P53 signaling pathway, and the JAK-STAT signaling pathway. The thresholds for GO enrichment were *p* < 0.05 and FDR < 0.15. The molecular functions of GO enrichment included transporter activity, ion transmembrane transporter activity, cation transmembrane transporter activity, gated channel activity, etc. The cellular components of GO enrichment included intrinsic components of the plasma membrane, tertiary granule membrane, cortical actin cytoskeleton, autophagosome, etc. The biological processes of GO enrichment included cation transport, positive regulation of antigen-receptor-mediated signaling pathways, transmembrane transport, etc. Bioinformatic analysis from the TCGA database was conducted to found out the expression relationship between SH3TC2 and MAP kinases. As shown in Figure S4, the expression of SH3TC2 was positively corelated with MAPK1, 3, 6, 7, 8, 9, 10, 11, 13, 14 and MAPK15. 

To further investigate the correlation between SH3TC2 and the MAPK pathway, we conducted qPCR analysis to determine the potential binding site of MAPK. As shown in Figure 5B, downregulation of SH3TC2 significantly inhibited the expression of MAPK8, 9, 13, and 14. As a result, our study implies that SH3TC2 may promote tumorigenesis via the MAPK pathway. 

### 3.6. Downregulated SH3TC2 Inhibited MAPK Pathways In Vitro

**Figure 6:**
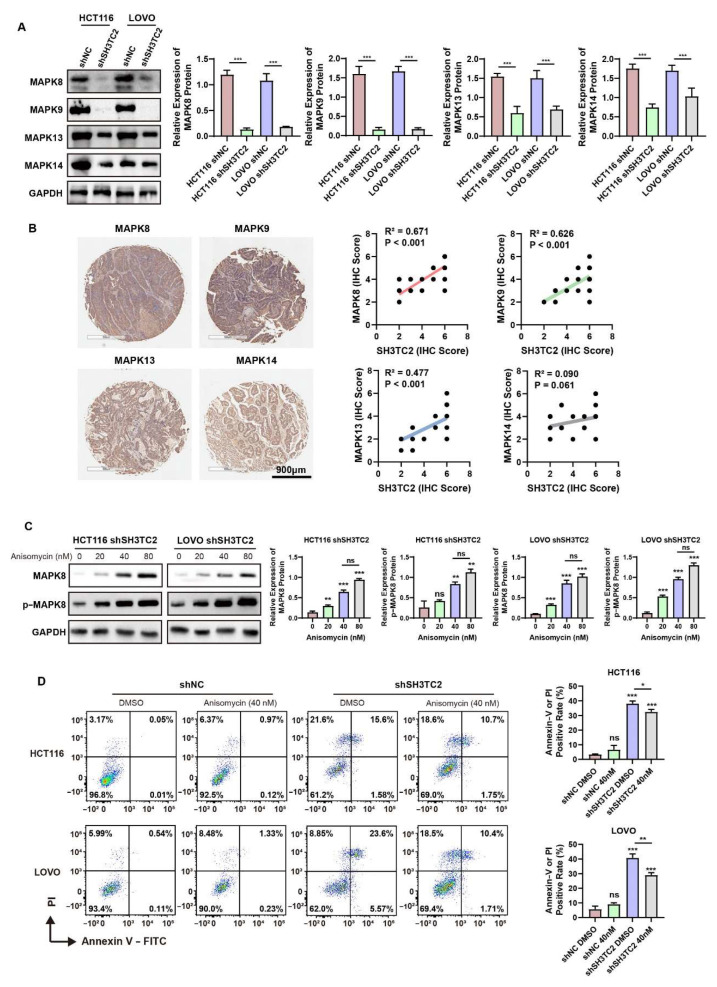
SH3TC2 knockdown regulates cell apoptosis via MAPK pathway. (**A**) Western blot analysis implied that MAPK8, 9, 13, and 14 were significantly inhibited after the stable knockdown of SH3TC2. (**B**) Analysis of clinical samples implied that expression of SH3TC2 was positively correlated with MAPK 8, 9, and 13. However, no statistical significance was found for MAPK14. (**C**) The implementation of MAPK8 activator significantly upregulated MAPK8 and p-MAPK8. (**D**) The rescue of MAPK8 partially reversed cell apoptosis of SH3TC2 stable knockdown cells. Unless specially marked specially, statistical analysis was compared to the blank or control group: ns, not significant; * *p* < 0.05; ** *p* < 0.01; *** *p* < 0.001.

In the previous figure, we screened that MAPK8, 9, 13, 14 would be downregulated after the stable knockdown of SH3TC2 in HCT116 and LOVO cell lines. To better understand the activities of the MAP kinases, Western blotting was utilized. As shown in Figure 6A, after the stable knockdown of SH3TC2, MAPK8, 9, 13, and 14 were significantly downregulated. The result implied that SH3TC2 may be a regulator for MAPK pathways. We also observed the relationship between SH3TC2 expression and MAP kinases in clinical specimens. As shown in Figure 6B, in CRC tumor specimens, SH3TC2 expression was positively correlated with expression of MAPK8, 9, and 13. However, no statistical significance was found for MAPK14. Due to the limited clinical specimens included in our study, a larger cohort should be included to further validate the conclusion. 

MAPK8 (also named c-Jun N-terminal kinase (JNK)) was significantly downregulated in the stable knockdown of SH3TC2 in CRC cell lines. The JNK activator Anisomycin was included in our study to further validate the function of SH3TC2. As shown in Figure 6C, with increasing concentrations of Anisomycin, MAPK8 and p-MAPK8 were significantly activated in SH3TC2 stable knockdown cells. Western blot revealed that changes in activity of MAPK8 and p-MAPK8 were not statistically significant between 40 nM and 80 nM Anisomycin. As a result, 40 nM Anisomycin was recruited for further analysis. The cell apoptosis experiment was utilized for rescue analysis. As shown in Figure 6D, the usage of 40 nM Anisomycin did not induce significant apoptosis in shNC cells, implying that usage of 40 nM Anisomycin was relatively non-toxic to cell lines. FACS analysis implied that the recovery of MAPK8 and p-MAPK8 activities would partially rescue the cells from apoptosis after the knockdown of SH3TC2. Our results display that knockdown of SH3TC2 may induce cell apoptosis via the MAPK8 pathway. However, after the recovery of MAPK8, cell viability could not be rescued to the previously level. The result implied that SH3TC2 may regulate cell viability via different pathways besides the MAPK8 pathway. 

### 3.7. Downregulation of SH3TC2 Inhibited Tumor Growth In Vivo

**Figure 7:**
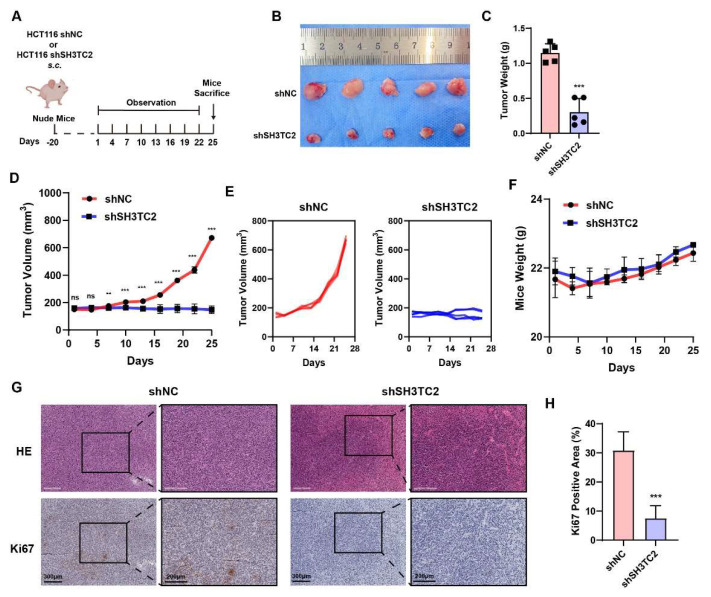
Knockdown of SH3TC2 inhibited tumor proliferation in vivo. (**A**) Scheme of the in vivo experiments. (**B**) Images of tumors resected from the mice at the endpoint. The tumor weight (**C**) and tumor volume (**D**) of the mice. (**E**) The spaghetti curves reveal that SH3TC2 silencing inhibited tumor proliferation in vivo. (**F**) The mouse weight did not change significantly during the experiment. (**G**) HE and Ki67 staining of the tumor tissue. (**H**) The Ki67-positive area in the negative control group was significantly higher than that in the shSH3TC2 group, indicating that silencing SH3TC2 inhibited tumorigenesis. Statistical analysis compared the two groups: ns, not significant; **, *p* < 0.01; ***, *p* < 0.001.

To establish the CRC model in nude mice, 5 × 10^6^ HCT116 negative control (HCT116 shNC) cells or HCT116 stable slicing SH3TC2 (HCT116 shSH3TC2) cells were subcutaneously injected into the right flank. The protocol of the in vivo study can be found in Figure 7A. At the end of in vivo experiments, the tumor was resected and stored at −80 °C. To identify the knockdown of SH3TC2 expression in vivo, IHC analysis was conducted. As shown in Figure S3, SH3TC2 expression was significantly downregulated in the shSH3TC2 group. Stable inhibition of SH3TC2 significantly limited the growth of the tumor, resulting in decreased tumor size (Figure 7B), tumor weight (Figure 7C), and tumor volume (Figure 7D). No difference was found in the mouse weights of the shSH3TC2 and shNC groups (Figure 7E). HE staining of the tumor tissue is shown in Figure 7G. The proliferation indicator (Ki67) IHC analysis revealed that the knockdown of SH3TC2 significantly downregulated tumor proliferation (Figure 7H).

## 4. Discussion

In previous research, the mutation of SH3TC2 has been considered a critical regulator for diseases of the central nervous system. Mutation or knockdown of SH3TC2 in mice results in peripheral neuropathy, resulting in decreased motor and sensory nerve conduction velocity and hypomyelination [52]. Recently, the role of SH3TC2 in tumor progression and metastasis has attracted the attention of researchers. Although SH3TC2 expression is prevalent in multiple cancer types and has been recently reported to play an important role in tumorigenesis and tumor progression, its prognostic value and molecular mechanism remain largely unclear.

In our present study, we analyzed the expression of SH3TC2 by using pan-cancer bioinformatic analysis. By mining the TCGA database, we found that SH3TC2 in tumor tissue was significantly overexpressed in most cancer types. However, SH3TC2 expression is downregulated in 12 types of cancer. The results indicate that SH3TC2 may act as an oncogene or tumor suppressor in different kinds of cancer. Its molecular mechanism still needs to be further explored. After integrating the analysis results in the GEPIA database, we found that SH3TC2 was significantly upregulated in COAD, READ, and SKCM, while SH3TC2 was downregulated in TGCT (Figure 1C). Moreover, higher expression of SH3TC2 was positively correlated with poor DFS of CRC patients (Figure 1D). These results indicate that SH3TC2 may play a critical regulatory role in CRC tumorigenesis. Hence, we further explored the role of SH3TC2 in CRC. After further analysis of the GEO datasets, we found that SH3TC2 in tumor tissue was significantly upregulated in CRC. Moreover, higher expression of SH3TC2 was found in locally advanced or metastatic CRC patients (Figure 2A). By analyzing the tumor immune microenvironment, we found that expression of SH3TC2 was correlated with T-cell infiltration. However, the results based on bioinformatic analysis were preliminary findings. The results should be further validated in clinical samples and further in vitro and in vivo experiments.

Although SH3TC2 is displayed as a prognostic factor across multiple tumor types via bioinformatic analysis, few studies have focused on the prognostic value of SH3TC2. In LAML, decreased expression of SH3TC2 was associated with MYCN amplification and poor survival [15]. Our study indicated that higher expression of SH3TC2 indicates poor DFS for CRC patients. However, expression of SH3TC2 did not correlate with the OS of CRC patients (Figure S1). Hence, higher expression of SH3TC2 may act as an indicator for tumor recurrence or metastasis but not for patient survival. However, due to the conflicting results of survival analysis, it is difficult to conclude that SH3TC2 would be a convincing prognostic indicator for CRC patients. More research with larger samples should be carried out to further validate the conclusion.

To further validate the role of SH3TC2 in CRC, we included 40 CRC patients and analyzed its expression via IHC analysis. In our cohorts, we found that SH3TC2 was significantly highly expressed in tumor tissue, and high expression also implied poor DFS. The in vivo study implied that downregulation of SH3TC2 significantly impaired tumorigenesis and tumor invasion and increased cell apoptosis.

In our research, we found that expression of SH3TC2 correlated with the MAPK pathway. The qPCR results implied that MAPK8, MAPK9, MAPK13, and MAPK14 were significantly downregulated after transfection with shSH3TC2. The results implied that SH3TC2 may regulate CRC tumorigenesis via the MAPK pathway. Furthermore, we validated the MAP kinases via Western blot assays. As shown in Figure 6, MAPK8 (also named JNK), MAPK9, MAPK13, and MAPK14 were significantly downregulated after stable knockdown of SH3TC2 in CRC cell lines, which was consistent with the qRT-PCR results. Moreover, we observed the positive expression correlation between MAPK8, 9, and 13 and SH3TC2 in clinical CRC cancer specimens. However, due to the limited number of CRC patients included in our study, a larger cohort with multicenter CRC patients should be enrolled in the future to further validate the results. 

The MAPK8 (JNK) activator Anisomycin was included in our study to further validate the function of SH3TC2. As shown in Figure 6C, with increasing concentration of Anisomycin, MAPK8 and p-MAPK8 were significantly activated in SH3TC2 stable knockdown cells. Cell apoptosis analysis implied that the recovery of MAPK8 and p-MAPK8 activities would partially rescue the cells from apoptosis after the knockdown of SH3TC2. Our results display that the knockdown of SH3TC2 may induce cell apoptosis via the MAPK8 pathway. However, after the recovery of MAPK8, cell viability could not be rescued to the previous level. The result implied that SH3TC2 may regulate cell viability via different pathways besides the MAPK8 pathway. 

JNK, also named MAPK8, is an important member of MAPK pathways, which play a vital role in regulating diverse cell processes [53]. It is increasing apparent that the persistent activation of JNK pathways may be involved in cancer development, progression, and metastasis [54]. Therefore, targeting JNKs by small molecular kinase inhibitors may be a novel therapeutic strategy for cancer treatments [53,54]. Researchers have found that the long noncoding RNA PVT1 may promote CRC progression by regulating the E2F3/MAPK8 pathways in CRC [55]. It has been reported that the MAPK pathways are located downstream of many growth-factor receptors, and overexpression and activation of MAPK may play an important role in CRC progression [56]. MAPK inhibitors for CRC treatment have also been introduced as clinical treatments [56,57]. However, emerging evidence has also shown a tumor suppressor role of JNK proteins [20,54,58]. The reason for the dual role of JNK or MAPK proteins may be different crossroads in the molecular pathways. As a result, the concrete molecular mechanism of JNK and MAPK pathways in CRC remain largely unknown. In our study, we observed that activating MAPK8 may reverse cell apoptosis after the knockdown of SH3TC2, and SH3TC2 knockdown may also downregulate MAPK8. Due to the complexity of the MAPK pathways in cancer research, the underlying molecular mechanism should be further studied in the future. 

Similar to the in vitro results, the in vivo study found that stable slicing of SH3TC2 significantly inhibited tumor growth in a CRC nude mouse model. Moreover, the knockdown of SH3TC2 may promote cancer cell apoptosis via multiple molecular mechanisms besides MAPK pathways. As a result, SH3TC2 may act as a therapeutic target for novel CRC treatment. For instance, delivering siRNAs by smart nanoparticles that target SH3TC2 may be a useful way to target CRC [59,60,61]. However, it is important to point out that mutation of SH3TC2 is closely related with diseases of the central nervous system [52]. We must admit that the design of small molecular–kinase inhibitors targeting SH3TC2 should pay attention to the potential harm to the central nervous system. 

## 5. Conclusions

In summary, through integrated bioinformatics analysis, we identified a novel oncogene, SH3TC2, for CRC. Expression of SH3TC2 was significantly upregulated in tumor tissue, which may imply poor DFS. To our knowledge, this is the first report that focuses on the molecular regulation and prognostic value of SH3TC2 in CRC. SH3TC2 may act as a novel oncogene for CRC. SH3TC2 expression may promote CRC tumor progression via the MAPK pathway. SH3TC2 inhibition impaired tumorigenesis both in vitro and in vivo. Overall, SH3TC2 may be a possible marker to optimize the prognosis of CRC and predict tumor recurrence and metastasis. SH3TC2 may act as a potential therapeutic target for CRC patients. However, larger CRC cohorts and experiments for further study are needed.

## Data Availability

The data presented in this study are available on request from the corresponding author.

## Supplementary Materials

The following supporting information can be downloaded at: https://www.mdpi.com/article/10.3390/cancers14153735/s1, Figure S1. The bioinformatic analysis of prognostic value of SH3TC2 in CRC. The bioinformatic analysis via GEPIA database indicated that the higher expression of SH3TC2 in tumor tissue implied poor DFS of CRC patients (including colon cancer or rectum cancer patients), while no statistical significance was found in OS. The DFS plot of CRC was taken from Figure 1D. Figure S2. The bi-oinformatic analysis of relative expression of SH3TC2 in CRC cell lines via CCLE database. Figure S3. The knockdown efficacy of SH3TC2 in shSH3TC2 group in vivo. Figure S4. The expression relationship between SH3TC2 and MAPKs via bioinformatic analysis in TCGA database. Figure S5. Source Western-blot Images for Figure 4A. The black, blue, and orange frame indicates three in-dependent experiments. The quantification analysis for the western-blot could be found in Table S4. Figure S6. Source Western-blot Images for Figure 4C. The black, blue, and orange frame indicates three independent siRNA transfection experiments. The quantification analysis for the western-blot could be found in Table S5. Figure S7. Source Western-blot Images for Figure 6A. The black, blue, and orange frame indicates three independent siRNA transfection experiments. The quantification analysis for the western-blot could be found in Table S7. Figure S8. Source Western-blot Images for Figure 6C. The black, blue, and orange frame indicates three independent siRNA transfection ex-periments. The quantification analysis for the western-blot could be found in Table S8. Table S1. Abbreviation of Cancer full Name in TCGA analysis. Table S2. The list of antibodies used in our study and dilution fold. Table S3. SH3TC2 siRNA sequences. Table S4. Primers used in qRT-PCR analysis. Table S5. The SH3TC2 expression and the clinical characteristic of CRC patients. Table S6. Source data for Figure 4A. Table S7. Source data for Figure 4C. Table S8. Source data for Figure 6A. Table S9. Source data for Figure 6C.
