# Supplementary material for: Pan-Cancer Analysis Reveals SH3TC2 as an Oncogene for Colorectal Cancer and Promotes Tumorigenesis via the MAPK Pathway
Source: Cancers (Basel). 2022 Jul 31;14(15):3735. doi: 10.3390/cancers14153735 (PMC9367385; doi:10.3390/cancers14153735)
Supplement: Supplementary file 1 [file cancers-14-03735-s001.zip › cancers-1801392-supplementary.pdf]

Supplementary Materials

Pan-Cancer Analysis Reveals SH3TC2 as an Oncogene for Colorectal Cancer and Promotes Tumorigenesis via the MAPK Pathway

Chengzhi Huang, Hui Yi, Yue Zhou, Qing Zhang and Xueqing Yao

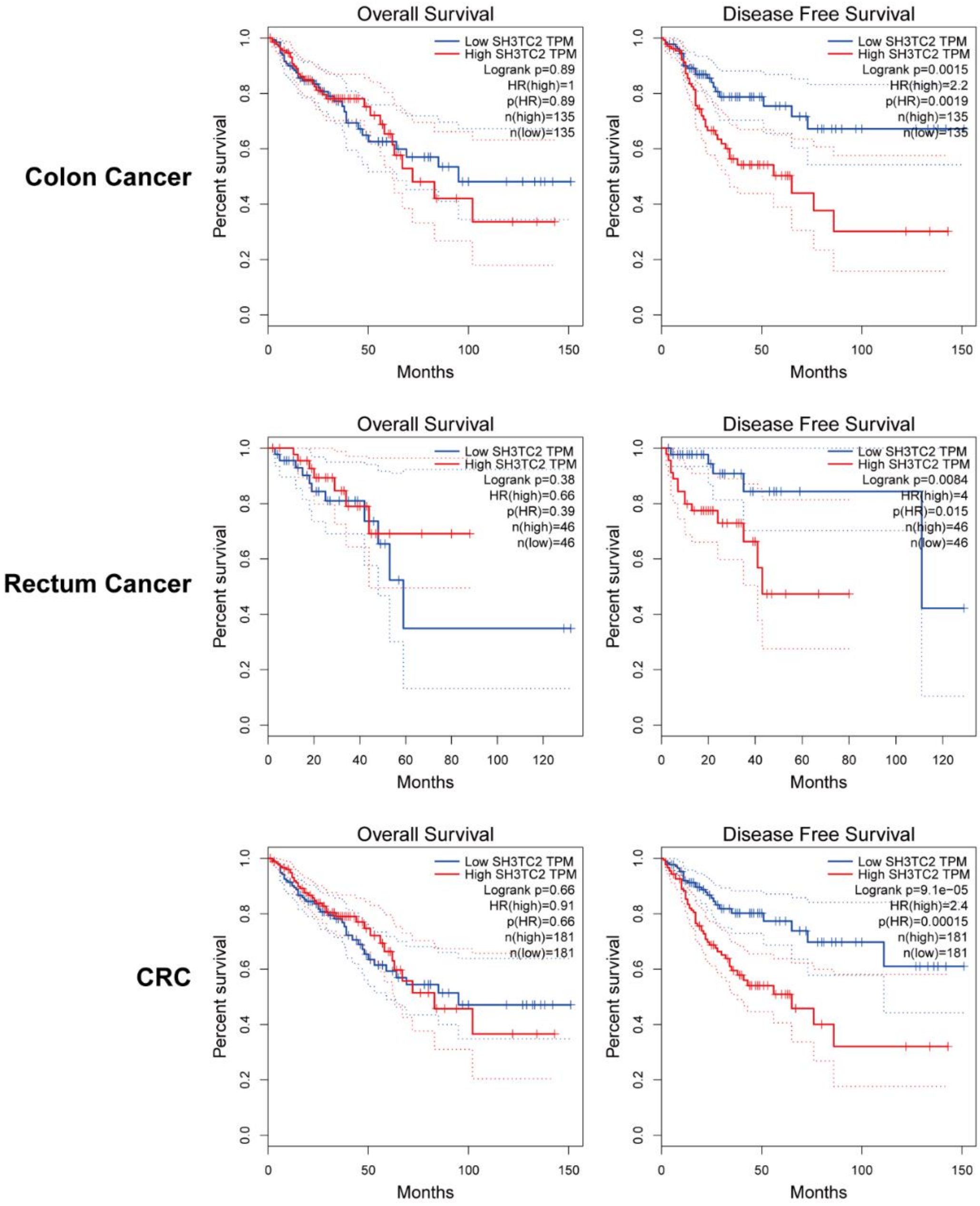

Figure S1. The bioinformatic analysis of prognostic value of SH3TC2 in CRC. The bioinformatic analysis via GEPIA database indicated that the higher expression of SH3TC2 in tumor tissue implied poor DFS of CRC patients (including colon cancer or rectum cancer patients), while no statistical significance was found in OS. The DFS plot of CRC was taken from Figure 1D.

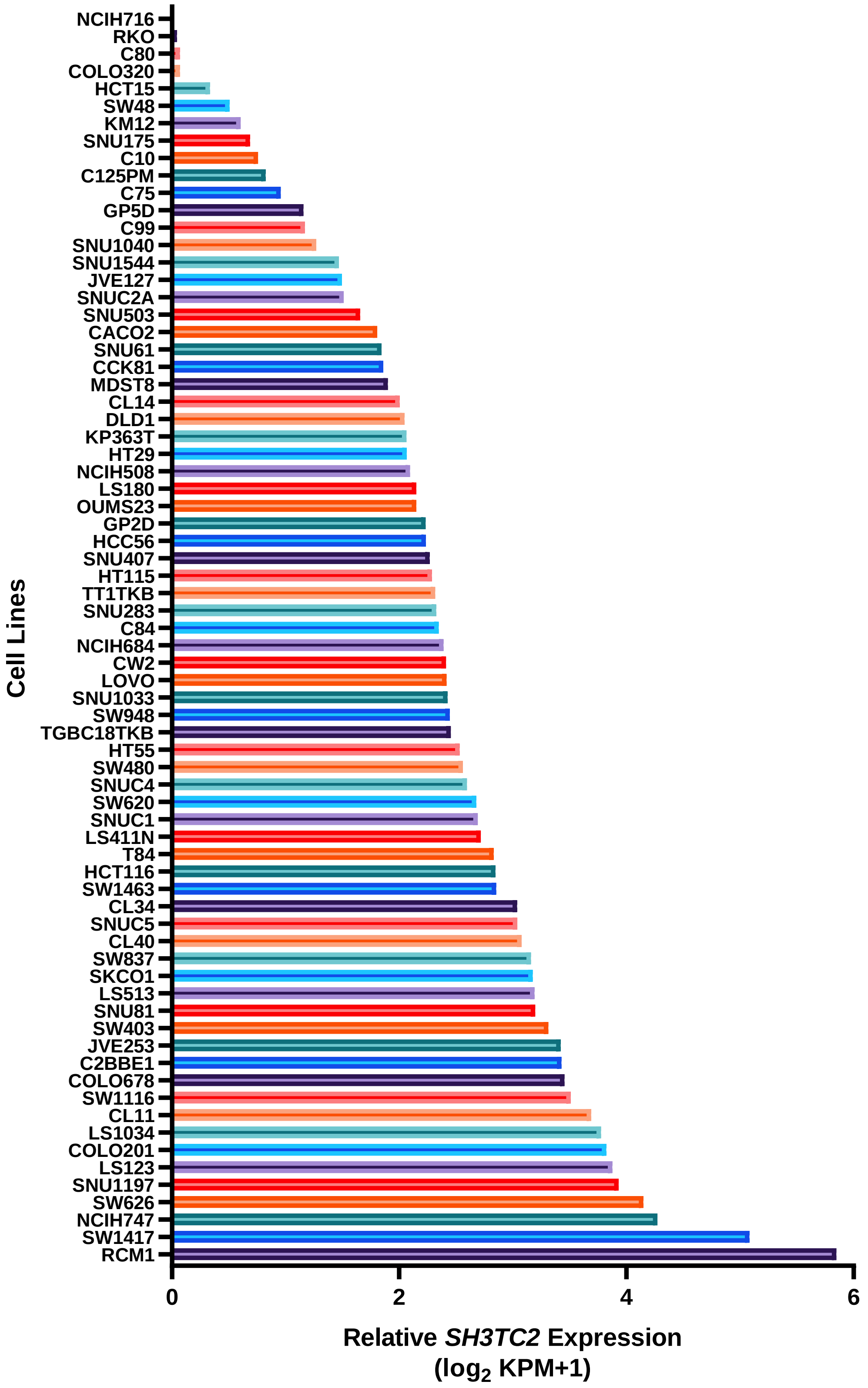

Figure S2. The bioinformatic analysis of relative expression of SH3TC2 in CRC cell lines via CCLE database.

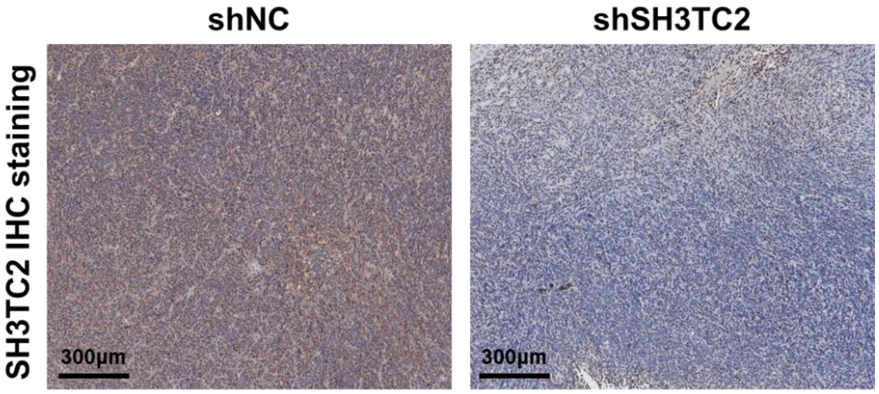

Figure S3. The knockdown efficacy of SH3TC2 in shSH3TC2 group in vivo.

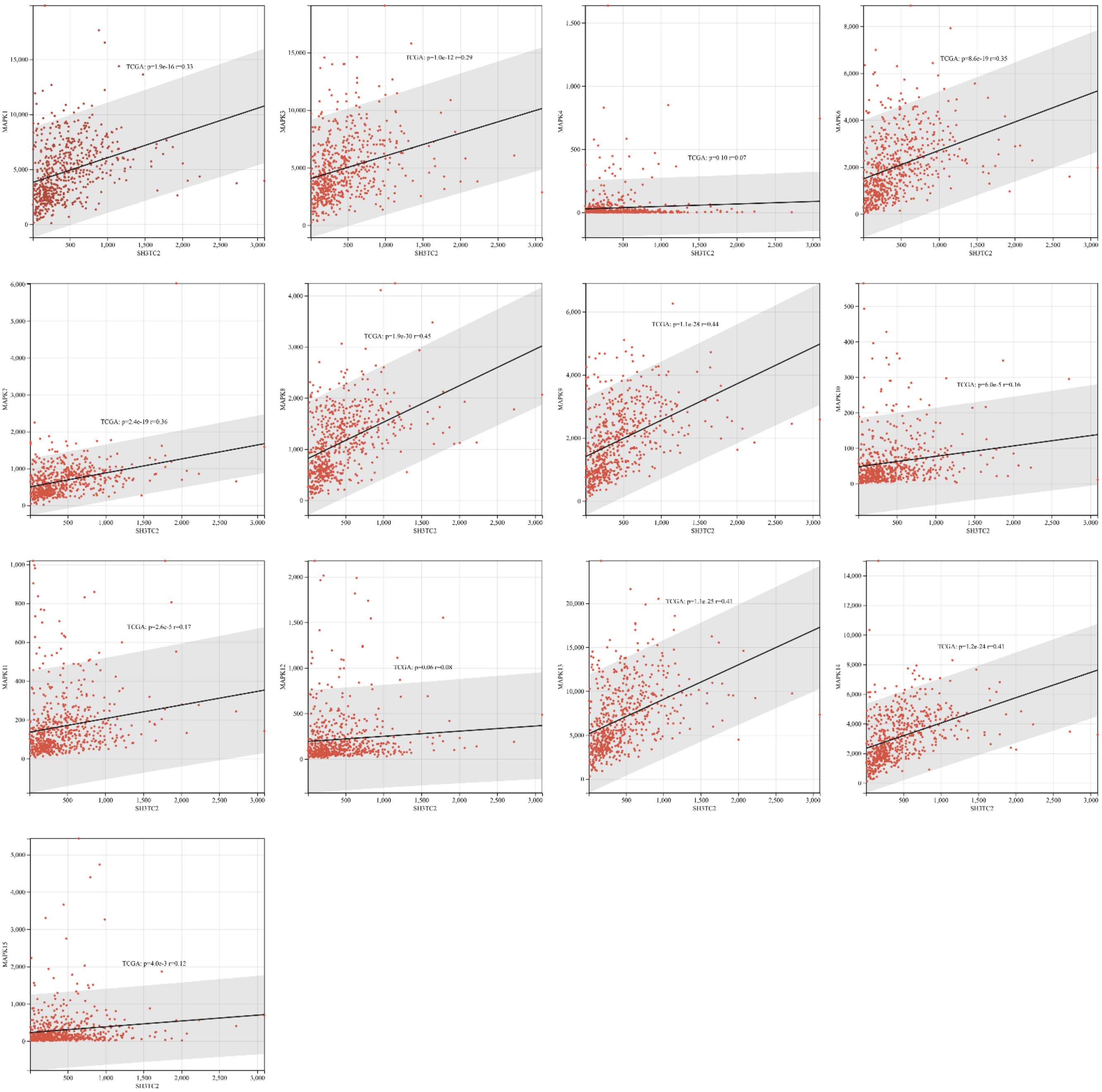

Figure S4. The expression relationship between SH3TC2 and MAPKs via bioinformatic analysis in TCGA database.

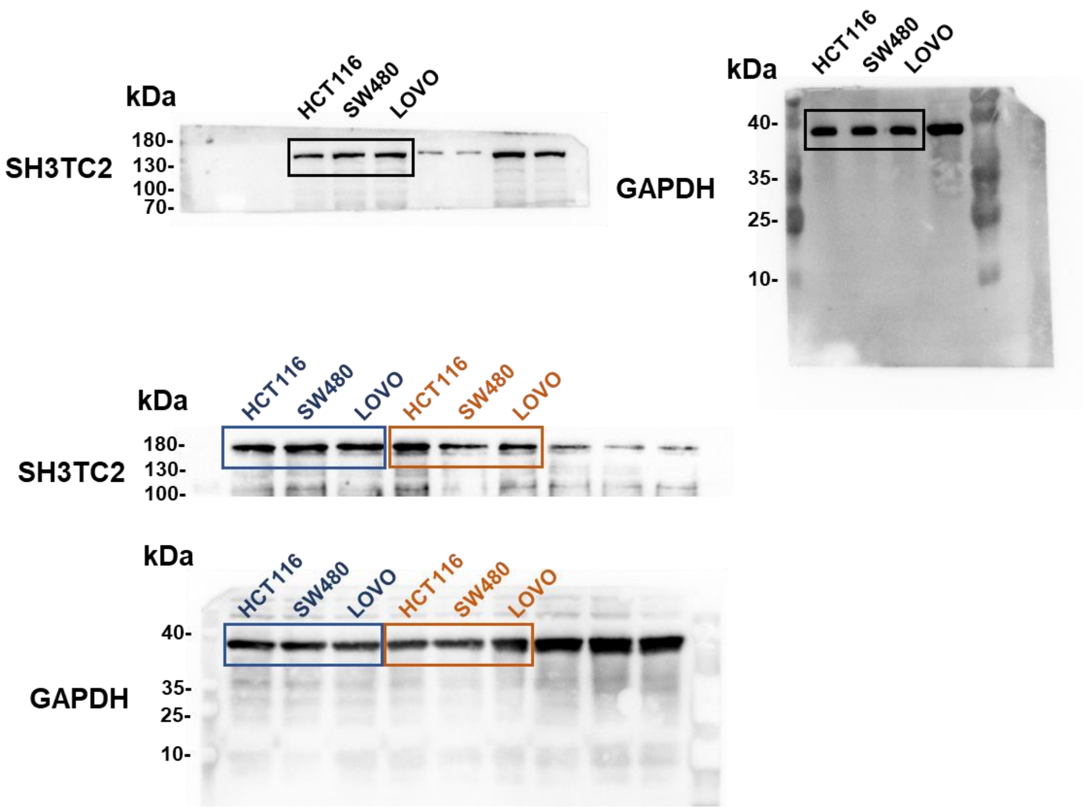

Figure S5. Source Western-blot Images for Figure 4A. The black, blue, and orange frame indicates three independent experiments. The quantification analysis for the western-blot could be found in Table S4.

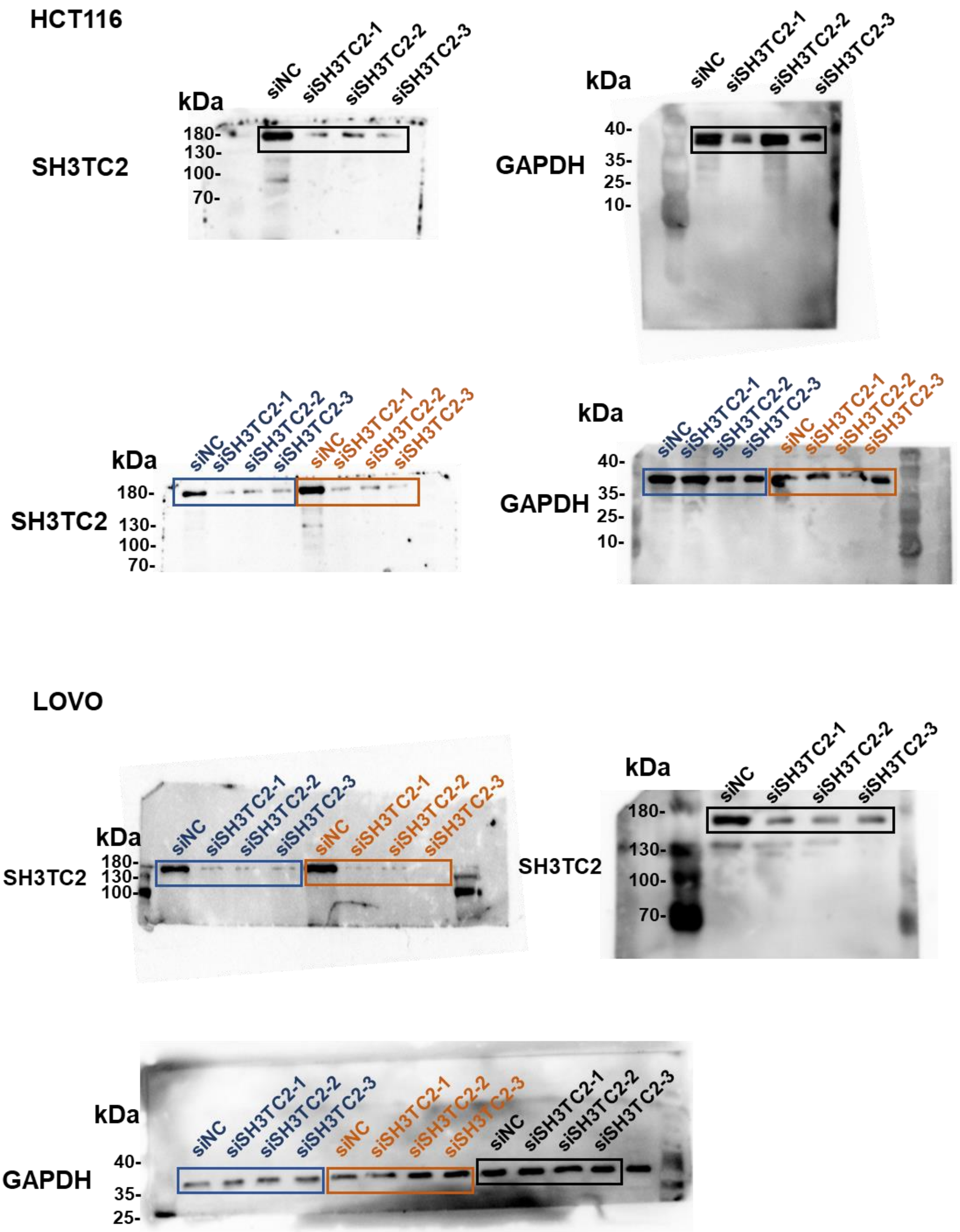

Figure S6. Source Western-blot Images for Figure 4C. The black, blue, and orange frame indicates three independent siRNA transfection experiments. The quantification analysis for the western-blot could be found in Table S5.

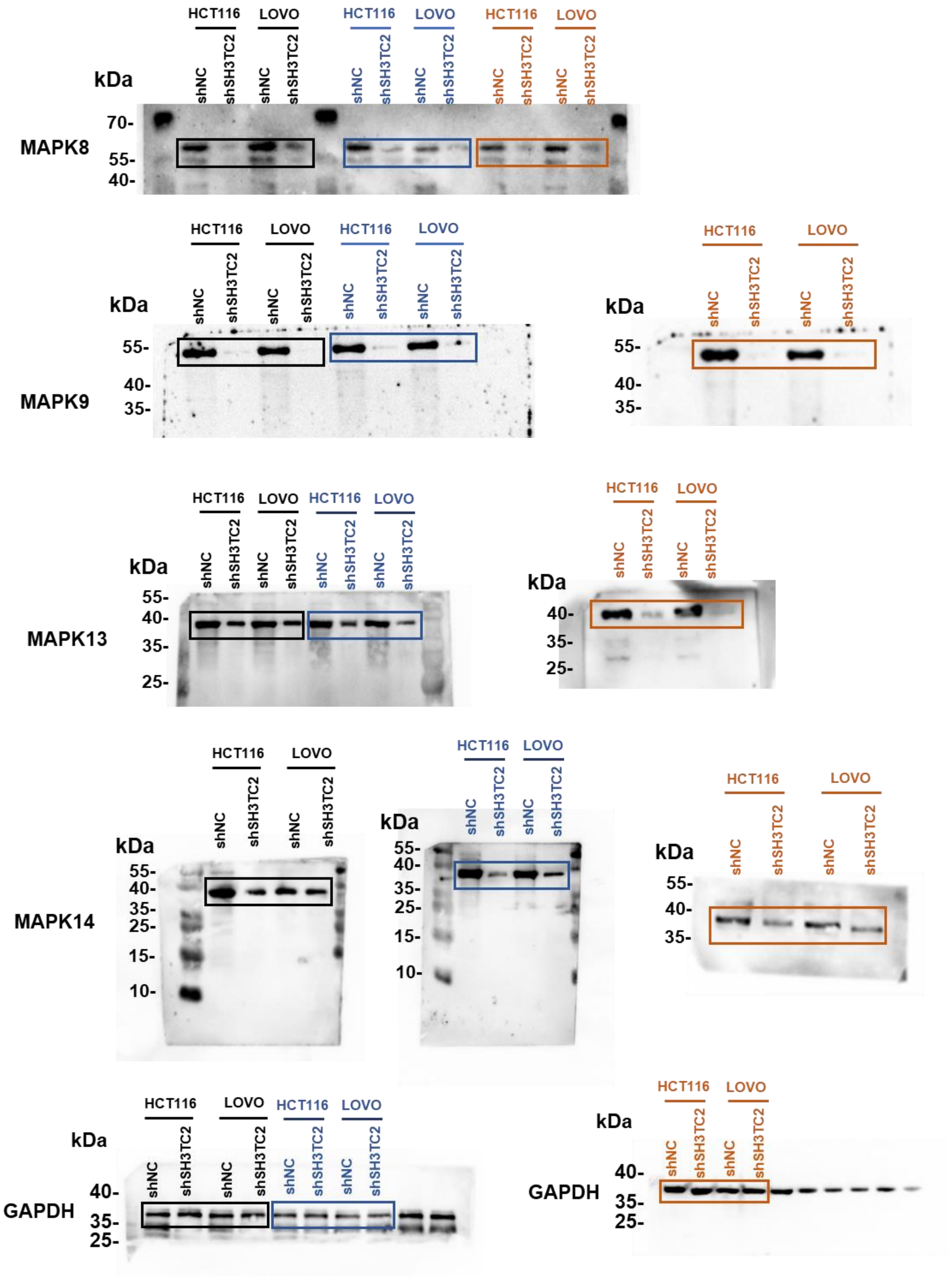

Figure S7. Source Western-blot Images for Figure 6A. The black, blue, and orange frame indicates three independent siRNA transfection experiments. The quantification analysis for the western-blot could be found in Table S7.

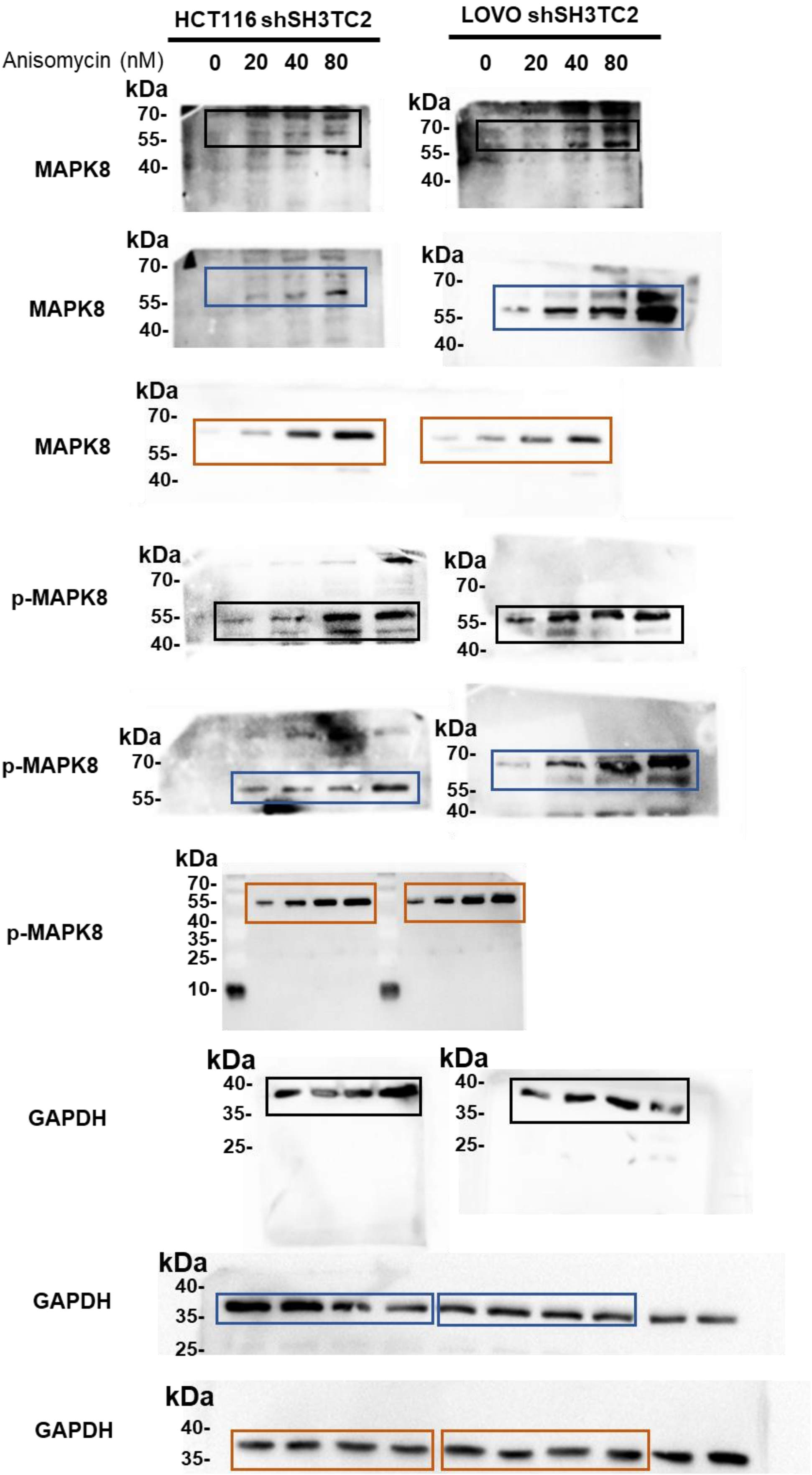

Figure S8. Source Western-blot Images for Figure 6C. The black, blue, and orange frame indicates three independent siRNA transfection experiments. The quantification analysis for the western-blot could be found in Table S8.

Table S1. Abbreviation of Cancer full Name in TCGA analysis.

Abbreviation	Full Name
ACC	Adrenocortical carcinoma
BLCA	Bladder Urothelial Carcinoma
BRCA	Breast invasive carcinoma
CESC	Cervical squamous cell carcinoma and endocervical adenocarcinoma
CHOL	Cholangiocarcinoma
COAD	Colon adenocarcinoma
COADREAD	Colon adenocarcinoma/Rectum adenocarcinoma Esophageal carcinoma
DLBC	Lymphoid Neoplasm Diffuse Large B-cell Lymphoma
ESCA	Esophageal carcinoma
FPPP	FFPE Pilot Phase II
GBM	Glioblastoma multiforme
GBMLGG	Glioma
HNSC	Head and Neck squamous cell carcinoma
KICH	Kidney Chromophobe
KIPAN	Pan-kidney cohort (KICH+KIRC+KIRP)
KIRC	Kidney renal clear cell carcinoma
KIRP	Kidney renal papillary cell carcinoma
LAML	Acute Myeloid Leukemia
LGG	Brain Lower Grade Glioma
LIHC	Liver hepatocellular carcinoma
LUAD	Lung adenocarcinoma
LUSC	Lung squamous cell carcinoma
MESO	Mesothelioma
OV	Ovarian serous cystadenocarcinoma
PAAD	Pancreatic adenocarcinoma
PCPG	Pheochromocytoma and Paraganglioma
PRAD	Prostate adenocarcinoma
READ	Rectum adenocarcinoma
SARC	Sarcoma
STAD	Stomach adenocarcinoma
SKCM	Skin Cutaneous Melanoma
STES	Stomach and Esophageal carcinoma
TGCT	Testicular Germ Cell Tumors
THCA	Thyroid carcinoma
THYM	Thymoma
UCEC	Uterine Corpus Endometrial Carcinoma
UCS	Uterine Carcinosarcoma
UVM	Uveal Melanoma

Table S2. The list of antibodies used in our study and dilution fold.

Antigen	Host	Manufacturer and Cat. No.	Dilution (x-fold) for Western-Blots	Dilution (x-fold) for IHC
SH3TC2	Rabbit	Bioss ; bs-11221R	1000	200
MAPK8 (JNK)	Mouse	Proteintech; 66210-1-Ig	3000	500
p-MAPK8 (p-JNK)	Rabbit	Proteintech; 80024-1-RR	1000	Not available
MAPK9	Rabbit	Sangon Biotech; D260527	1000	200
MAPK13	Rabbit	Sangon Biotech; D160920	1000	200
MAPK14	Mouse	Proteintech; 66234-1-Ig	1000	200
GAPDH	Rabbit	Bioworld; AP0066	5000	Not available
HRP-conjugated anti-rabbit secondary antibody	Goat	Bioworld; BS13278	5000	Not available
HRP-conjugated anti-mouse secondary antibody	Goat	Proteintech; SA00001-1	5000	Not available

Table S3. SH3TC2 siRNA sequences.

Name		SH3TC2 RNA Oligo Sequences (5' - 3')
siSH3TC2-1	Guide	UUCUUCUUGUGAAAUGAGGGG
	Passenger	CCUCAUUUCACAAGAAGAAGA
siSH3TC2-2	Guide	UUUUCAGAGGCUGUUUGUGUG
	Passenger	CACAAACAGCCUCUGAAAAGC
siSH3TC2-3	Guide	UUAUGUAUGGAGAAAGUGCUU
	Passenger	GCACUUUCUCCAUACAUAAUU

Table S4. Primers used in qRT-PCR analysis.

Target		Primer Sequences (5' - 3')
SH3TC2	Forward	CTGTAAGGCCTTGACGGGTT
	Reverse	GAGTTCCTGCTCATTGGGGA
MAPK1	Forward	CAGTTCTTGACCCCTGGTCC
	Reverse	TACATACTGCCGCAGGTCAC
MAPK3	Forward	GCCAAGCTTTTCCCCAAGTC
	Reverse	CAGCCGCTCCTTAGGTAGGT
MAPK4	Forward	GCTCTGCCTCGGGAAGGA
	Reverse	GCATCCAGGGGCTTCTTTCT
MAPK6	Forward	CGTGGAGTCTCGCCTTACTG
	Reverse	ACTTCAGGGCTCTGAGACGA
MAPK7	Forward	CAAGAACCTGGCCCTGCTTA
	Reverse	CAGGACCACGTAGACAGATTTGA
MAPK8	Forward	CTGAAGCAGAAGCTCCACCA
	Reverse	GCCATTGATCACTGCTGCAC
MAPK9	Forward	AGTCATCCTGGGTATGGGCT
	Reverse	GGAAGGATACGGTCAGTGCC
MAPK10	Forward	AGAGGGTGATTCAGCAAGCC
	Reverse	TTTGCCCCAGAAATCCTCCC
MAPK11	Forward	GAACAAGACCGTGTGGGAGG
	Reverse	GGAATTGAACGTGCTCGTCG
MAPK12	Forward	GGCTGAGGTATATCCACGCTG
	Reverse	AACAGCGTCTTGCCTGTGAT
MAPK13	Forward	CCCTCTGTGGGCAGCATTTA
	Reverse	CAGCTCAAAGGTACAGCCCA
MAPK14	Forward	GCATAATGGCCGAGCTGTTG
	Reverse	TCATGGCTTGGCATCCTGTT
MAPK15	Forward	AGATACCTACTCAGGCGGCA
	Reverse	GATGGTCCCCAAACTCCTGG
GAPDH	Forward	TCAACGGATTTGGTCGTATTGGGCG
	Reverse	CTCGCTCCTGGAAGATGGTGATGGG

Table S5. The SH3TC2 expression and the clinical characteristic of CRC patients.

Group	*n*	SH3TC2 Protein Expression in IHC analysis			
		Low expression	High Expression	Chi-squre	P
Total	40	20	20		
**Gender**					
Female	28	15	13	0.4762	0.490
Male	12	5	7		
**Age**					
≤60	9	4	5	0.1434	0.705
>60	31	16	15		
**Tumor Location**					
Colon	24	13	11	0.4167	0.519
Rectum	16	7	9		
**Tumor Stage (TNM Stage)**					
I + II	17	14	3	12.3785	<0.001
III + IV	23	6	17		
**Tumor Differentiation**					
Median or Well	31	16	15	0.1434	0.705
Poor	9	4	5		

Table S6. Source data for Figure 4A.

Target	Sample	Relative Grey Density 1	Relative Grey Density 2	Relative Grey Density 3	Relative Expression 1	Relative Expression 2	Relative Expression 3
SH3TC2	HCT116	44850	106975	89042	0.85	0.87	1.09
	SW480	47906	107437	88259	0.86	1.00	0.88
	LOVO	51333	100246	75782	0.97	1.08	0.64
GAPDH	HCT116	52967	123107	81748			
	SW480	55474	106952	99760			
	LOVO	52701	92790	117603			

Table S7. Source data for Figure 4C.

Cell Line	Target	Sample	Relative Grey Density 1	Relative Grey Density 2	Relative Grey Density 3	Relative Expression 1	Relative Expression 2	Relative Expression 3
HCT116	SH3TC2	siNC	90866	101831	65023	0.84	0.97	1.06
		siSH3TC2-1	10114	10005	9764	0.20	0.10	0.24
		siSH3TC2-2	10906	11141	10147	0.12	0.20	0.23
		siSH3TC2-3	9728	11507	5386	0.18	0.20	0.12
	GAPDH	siNC	108031	104683	61210			
		siSH3TC2-1	49561	97063	40406			
		siSH3TC2-2	91892	55013	43335			
		siSH3TC2-3	55281	57489	44271			
LOVO	SH3TC2	siNC	75030	85550	83401	1.05	0.94	0.98
		siSH3TC2-1	18097	11390	11615	0.23	0.13	0.14
		siSH3TC2-2	10927	16166	17132	0.15	0.17	0.21
		siSH3TC2-3	16786	14159	10337	0.22	0.15	0.12
	GAPDH	siNC	71661	91010	84905			
		siSH3TC2-1	78413	90188	82675			
		siSH3TC2-2	71687	93372	82536			
		siSH3TC2-3	75353	94171	85157			

Table S8. Source data for Figure 6A.

Target	Sample	Relative Grey Density 1	Relative Grey Density 2	Relative Grey Density 3	Relative Expression 1	Relative Expression 2	Relative Expression 3
MAPK8	HCT116 shNC	76768	65188	65862	1.2818167	1.1976484	1.1128157
	HCT116 shSH3TC2	8808	8460	8479	0.1048846	0.1429972	0.1484523
	LOVO shNC	79753	51060	52006	1.2351019	1.0096097	1.0010972
	LOVO shSH3TC2	13664	7599	9960	0.1854632	0.1882525	0.1719346
MAPK9	HCT116 shNC	100553	140590	134955	1.6789614	1.7494369	1.3969485
	HCT116 shSH3TC2	9675	9388	3521	0.1152087	0.214245	0.151931
	LOVO shNC	109562	152143	122086	1.6967416	1.7806165	1.5366587
	LOVO shSH3TC2	9599	5905	7511	0.1302884	0.1852956	0.196824
MAPK13	HCT116 shNC	91866	80363	96607	1.5339122	1.4764468	1.6322886
	HCT116 shSH3TC2	54455	43819	23175	0.6484436	0.7406612	0.4057532
	LOVO shNC	83991	85444	79449	1.3007341	1.6894847	1.5293653
	LOVO shSH3TC2	47110	31868	38161	0.6394299	0.7894763	0.6587547
MAPK14	HCT116 shNC	107868	100122	96848	1.801102	1.8394635	1.6363606
	HCT116 shSH3TC2	62547	39236	47685	0.7448022	0.663196	0.8348799
	LOVO shNC	109278	79363	95752	1.6923434	1.5692451	1.8431924
	LOVO shSH3TC2	68457	51629	52140	0.9291754	1.2790219	0.9000673
GAPDH	HCT116 shNC	59890	54430	59185			
	HCT116 shSH3TC2	83978	59162	57116			
	LOVO shNC	64572	50574	51949			
	LOVO shSH3TC2	73675	40366	57929			

Table S9. Source data for Figure 6C.

Cell Line	Target	Anisomycin Concentration (nM)	Relative Grey Density 1	Relative Grey Density 2	Relative Grey Density 3	Relative Expression 1	Relative Expression 2	Relative Expression 3
HCT116 shSH3TC2	MAPK8	0	16098	12033	13994	0.171372	0.1139672	0.1338178
		20	31403	29517	29802	0.3220556	0.286634	0.2804182
		40	64922	66266	64790	0.6878423	0.6455214	0.5969008
		80	94336	91230	96450	0.9540743	0.9108245	0.9644228
HCT116 shSH3TC2	p-MAPK8	0	14638	19831	46211	0.1558295	0.1878238	0.4418934
		20	43348	43911	41988	0.4445584	0.4264115	0.3950808
		40	84160	81418	89478	0.8916671	0.7931226	0.8243477
		80	118863	112671	104699	1.2021299	1.1248877	1.0469062
HCT116 shSH3TC2	GAPDH	0	93936	105583	104575			
		20	97508	102978	106277			
		40	94385	102655	108544			
		80	98877	100162	100008			
LOVO shSH3TC2	MAPK8	0	10553	11848	10679	0.1038927	0.1048319	0.1012535
		20	35675	35195	32024	0.3485589	0.3174983	0.311112
		40	79562	102312	98788	0.7608055	0.8969229	0.9006519
		80	95990	109606	110146	0.9497284	1.0705593	1.0419737
LOVO shSH3TC2	p-MAPK8	0	11365	11670	15804	0.1118867	0.103257	0.1498464
		20	57762	56539	53065	0.5643576	0.510045	0.5155245
		40	105479	106605	101824	1.0086349	0.9345577	0.9283311
		80	135198	135755	130730	1.3376537	1.3259655	1.236697
LOVO shSH3TC2	GAPDH	0	101576	113019	105468			
		20	102350	110851	102934			
		40	104576	114070	109685			
		80	101071	102382	105709			
